# Evolution and Management of Cognitive Disorders Related to Chronic Cannabis Use: A Literature Review

**DOI:** 10.1192/j.eurpsy.2026.10498

**Published:** 2026-07-31

**Authors:** H. Hsine, W. Abbes, S. Kolsi, A. Bouaziz

**Affiliations:** 1 Psychiatry, La Métairie Clinic, Nyon, Switzerland; 2 Psychiatry, University Hospital of Gabès, Gabes, Tunisia

## Abstract

**Introduction:**

Cognitive disorders associated with chronic cannabis use represent a frequent complaint in clinical practice, particularly among young adults. While the deleterious effects of this substance on memory, attention, and executive functions are well documented, the question of their evolution after cessation remains debated.

**Objectives:**

This review aims to examine the progression of cognitive disorders after cannabis withdrawal, as well as the available therapeutic strategies.

**Methods:**

A systematic literature review, following the PRISMA-P methodology, was conducted across PubMed, PsycINFO, and Cochrane Library databases between 2014 and 2024. Of the 30 articles included, 9 specifically examined cognitive outcomes post-abstinence and management strategies.

**Results:**

Cannabis cessation is associated with partial or complete improvement of cognitive functions after 4 weeks, particularly in verbal memory. However, some deficits persist. Regarding management, pharmacological treatments (cannabidiol, acetylcholinesterase inhibitors) did not demonstrate clear benefits. In contrast, non-pharmacological approaches, such as cognitive-behavioral therapy (CBT), cognitive remediation, and Approach Bias Modification, appear promising.

**Conclusions:**

The evolution of cognitive disorders after cannabis cessation is generally favorable. Tailored cognitive and behavioral strategies can enhance this recovery.

**Disclosure of Interest:**

None Declared

